# Aqua­{4,4′-dimeth­oxy-2,2′-[pyridine-2,3-diylbis(nitrilo­methanylyl­idene)]­diphenolato}copper(II)

**DOI:** 10.1107/S1600536812031273

**Published:** 2012-07-14

**Authors:** Fatiha Benghanem, Razika Benramdane, Sofiane Bouacida, Saida Keraghel, Ali Ourari

**Affiliations:** a Laboratoire d’Electrochimie, d’Ingénierie Moléculaire et de Catalyse Redox (LEIMCR), Faculté des Sciences de l’Ingénieur, Université Farhat Abbas, Sétif 19000, Algeria; b Unité de Recherche de Chimie de l’Environnement et Moléculaire Structurale (CHEMS), Université Mentouri–Constantine 25000, Algeria

## Abstract

Mol­ecules of the title compound, [Cu(C_21_H_17_N_3_O_4_)(H_2_O)], lie across a crystallographic mirror plane. The Cu^II^ atom is five-coordinated in a distorted square-pyramidal environment by two phenolate O atoms and two imine N atoms of the tetra­dentate Schiff base anion in the basal plane and one water mol­ecule in the apical position. Because of symmetry, the pyridine N atom and the corresponding C atom at the 4-position of the pyridine ring are disordered. The crystal packing can be described as being composed of alternating layers stacked along [001]. Intra­molecular C—H⋯N and inter­molecular C—H⋯O and O—H⋯O hydrogen-bonding inter­actions, as well as C—H⋯π and π–π stacking inter­actions [shortest centroid–centroid distance = 3.799 (8) Å and inter­planar distance = 3.469 (2) Å] are observed.

## Related literature

For background, see Ourari *et al.* (2006 ▶); Ouari *et al.* (2010 ▶); Ourari, Ouari *et al.* (2008 ▶); Vyas & Shah (1963 ▶); Kataoka *et al.* (1979 ▶). For applications, see: Ourari, Baameur *et al.* (2008 ▶); Coche-Guerente *et al.* (1995 ▶). For the synthesis, see: Huo *et al.* (1999 ▶); Khedkar & Radhakrishnan (1997 ▶); Guo & Wong (1999 ▶).
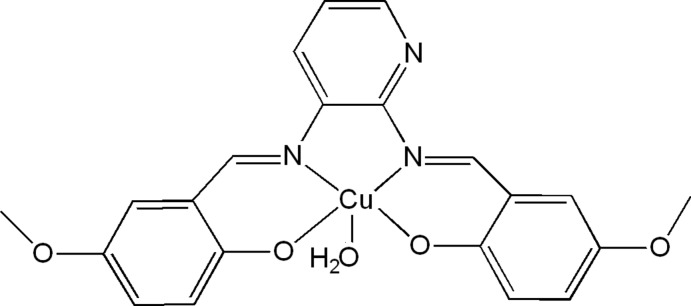


## Experimental

### 

#### Crystal data

[Cu(C_21_H_17_N_3_O_4_)(H_2_O)]
*M* *_r_* = 456.93
Orthorhombic, 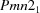

*a* = 23.162 (7) Å
*b* = 5.0997 (14) Å
*c* = 7.769 (2) Å
*V* = 917.7 (4) Å^3^
*Z* = 2
Mo *K*α radiation
μ = 1.23 mm^−1^
*T* = 296 K
0.12 × 0.06 × 0.04 mm

#### Data collection

Bruker APEXII CCD diffractometer
10830 measured reflections
3026 independent reflections
2384 reflections with *I* > 2σ(*I*)
*R*_int_ = 0.056

#### Refinement

*R*[*F* ^2^ > 2σ(*F* ^2^)] = 0.032
*wR*(*F* ^2^) = 0.060
*S* = 0.89
3026 reflections
142 parameters
2 restraints
H atoms treated by a mixture of independent and constrained refinement
Δρ_max_ = 0.28 e Å^−3^
Δρ_min_ = −0.42 e Å^−3^
Absolute structure: Flack (1983 ▶), 1361 Friedel pairs
Flack parameter: −0.015 (11)

### 

Data collection: *APEX2* (Bruker, 2001 ▶); cell refinement: *SAINT* (Bruker, 2001 ▶); data reduction: *SAINT*; program(s) used to solve structure: *SIR2002* (Burla *et al.*, 2005 ▶); program(s) used to refine structure: *SHELXL97* (Sheldrick, 2008 ▶); molecular graphics: *ORTEP-3 for Windows* (Farrugia, 1997 ▶) and *DIAMOND* (Brandenburg & Berndt, 2001 ▶); software used to prepare material for publication: *WinGX* (Farrugia, 1999 ▶).

## Supplementary Material

Crystal structure: contains datablock(s) global, I. DOI: 10.1107/S1600536812031273/wm2656sup1.cif

Structure factors: contains datablock(s) I. DOI: 10.1107/S1600536812031273/wm2656Isup2.hkl

Additional supplementary materials:  crystallographic information; 3D view; checkCIF report

## Figures and Tables

**Table 1:** Selected bond lengths (Å)

Cu1—O1	1.9126 (14)
Cu1—N1	1.9561 (16)
Cu1—O3	2.416 (3)

**Table 2:** Hydrogen-bond geometry (Å, °) *Cg* is the centroid of the C2–C7 benzene ring.

*D*—H⋯*A*	*D*—H	H⋯*A*	*D*⋯*A*	*D*—H⋯*A*
O3—H1*W*⋯O1^i^	0.84 (2)	2.19 (2)	2.933 (3)	146 (2)
C8—H8*A*⋯O2^ii^	0.93	2.57	3.330 (3)	139
C8—H8*A*⋯N2	0.93	2.49	2.844 (3)	103
C1—H1*B*⋯*Cg* ^iii^	0.96	2.71	3.528 (4)	143

Symmetry codes: (i) 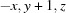; (ii) 
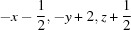
; (iii) 
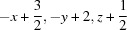
.

**Fig. 1.**
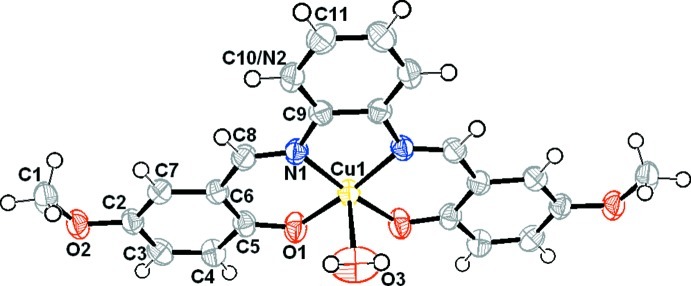
The molecular geometry of (I) with the atom-labelling scheme. Displacement ellipsoids are drawn at the 50% probability level. H atoms are represented as small spheres of arbitrary radius. Only the contents of the asymmetric unit are numbered.

**Fig. 2.**
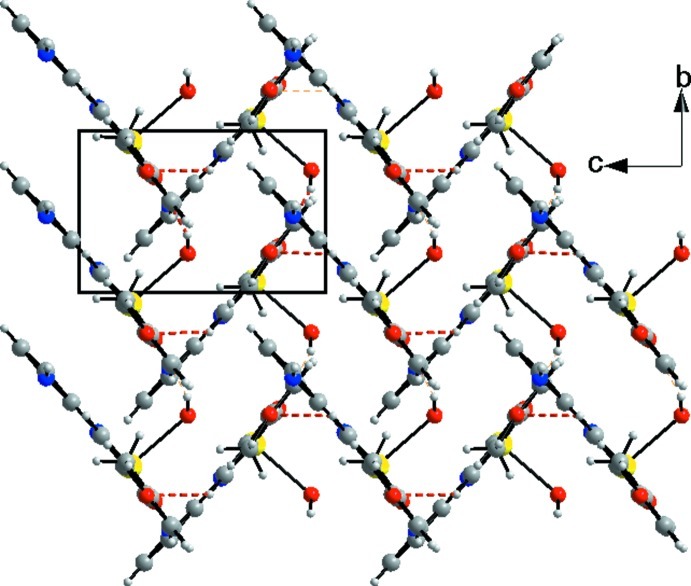
The crystal packing of (I) viewed along the a axis showing alterning zigzag layers.

**Fig. 3.**
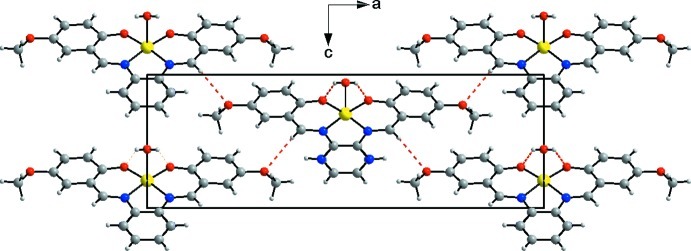
The crystal packing of (I) viewed along the b axis showing intermolecular hydrogen bonding interactions [C—H···O and O—H···O] as dashed lines.

**Fig. 4.**
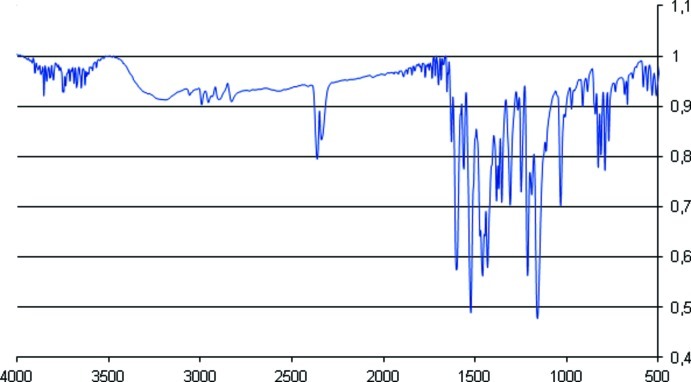
The infrared spectrum of the title complex (I), attesting the presence of water.

## supplementary crystallographic information

### Comment

The title compound is one of the targeted materials to elaborate modified electrodes in order to use them in heterogeneous electrocatalysis. Therefore, this work is a continuation of investigations where 2,3-diaminophenol and 2,3-diaminopyridine were involved (Ourari *et al.*, 2006; Ouari *et al.*, 2010; Ourari, Ouari *et al.*, 2008). The resulting ligands or complexes may be functionalized by etherification (Vyas & Shah, 1963) or quaternization (Kataoka *et al.*, 1979) reactions using *N*-(3-bromopropyl)pyrrole. These materials are mainly applied in catalysis, electrocatalysis and sensors (Ourari, Baameur *et al.*, 2008; Coche-Guerente *et al.*, 1995). The synthesis of new salicylaldehyde derivatives containing electropolymerizable units can be considered as the main source of functionalized π-conjugated conducting polymers as those of poly(pyrrole), poly(thiophene) and poly(aniline) (Huo *et al.*, 1999; Khedkar & Radhakrishnan, 1997; Guo & Wong, 1999). We report here the synthesis of the title compound, [Cu(C_21_H_17_N_3_O_4_)(H_2_O)], (I) and its crystal structure.

The molecular geometry of (I), and the atomic numbering used, is illustrated in Fig. 1. The asymmetric unit of (I) consists of one-half of the molecule, with the other half generated by a crystallographic mirror plane. Due to this symmetry the N2 and C10 atoms of the pyridine ring are equally disordered. The Cu^II^ atom is five-coordinate in a distorted square pyramidal geometry by the O atoms of two 5-methoxysalicylidene groups, the imine N atoms and one molecule of water in the apical position. The bond lengths of the coordination sphere range from 1.9126 (14) to 2.416 (3) Å for Cu—O distances and is 1.9561 (16) Å for the Cu—N distance. The crystal packing in (I) can be described by alterning layers along [001] (Fig. 2). There are one intramolecular C—H···N and two intermolecular C—H···O and O—H···O hydrogen bonding interactions in this packing (Fig. 3), which is further stabilized by C—H···π interactions (Table 2) and π–π stacking (shortest centroid-to-centroid distance 3.799 (8); interplanar distance of 3.469 (2) Å).

### Experimental

All reagents were obtained from commercial sources and used without any further purification. 59 mg (0.3 mmol) of copper acetate monohydrate were dissolved in MeOH (10 ml). This solution was added dropwise to a stirred methanolic solution (5 ml) containing 113 mg (0.3 mmol) of the Schiff base ligand (*N*,*N*-bis(5-Methoxysalicyidene)-2,3-diaminopyridine). This mixture was stirred and refluxed for 90 minutes under nitrogen atmosphere to give a brown precipitate which was collected by filtration and washed successively with methanol and diethyl ether. After drying in vacuum in the presence of CaCl_2_, 93.5 mg of the copper complex were obtained (71%). Suitable crystals of green color were obtained from the filtrate after about twenty days. The microanalysis of (I) showed that one molecule of water is present (calc. / found %: C:55.20 /54.78; H:04.19 / 04.12; N: 09.20 / 09.17). Moreover, analysis by infrared spectrometry showing a spectrum with an absorption band at 1628 cm^-1^ attesting the presence of lattice water with its characteristic vibration band (H—O—H bending) accompanied by the stretching band at about 3500 cm^-1^ (Fig. 4).

### Refinement

H atoms were localized in Fourier difference maps but introduced in calculated positions and treated as riding on their parent atom (C) with C—H = 0.93 Å (methine), 0.96 Å (methyl), and 0.93 Å (aromatic) with *U*_iso_(H) = 1.2*U*_eq_(C_aromatic_ and C_methine_) and *U*_iso_(H) = 1.5*U*_eq_(C_methyl_). The H1W proton of the water molecule was located in a difference Fourier map and refined isotropically with *U*_iso_(H) = 1.5*U*_eq_(O). Atoms N2 and C10 (with the attached proton) are disordered due to symmetry and were refined with a 0.5 occupancy each.

### Crystal data

**Table:**

[Cu(C_21_H_17_N_3_O_4_)(H_2_O)]	*F*(000) = 470
*M**_r_* = 456.93	*D*_x_ = 1.654 Mg m^−^^3^
Orthorhombic, *P**m**n*2_1_	Mo *K*α radiation, λ = 0.71073 Å
Hall symbol: P 2ac -2	Cell parameters from 2879 reflections
*a* = 23.162 (7) Å	θ = 2.8–24.8°
*b* = 5.0997 (14) Å	µ = 1.23 mm^−^^1^
*c* = 7.769 (2) Å	*T* = 296 K
*V* = 917.7 (4) Å^3^	Prismatic, green
*Z* = 2	0.12 × 0.06 × 0.04 mm

### Data collection

**Table:**

Bruker APEXII CCD diffractometer	2384 reflections with *I* > 2σ(*I*)
Radiation source: fine-focus sealed tube	*R*_int_ = 0.056
Graphite monochromator	θ_max_ = 31.5°, θ_min_ = 2.8°
φ and ω scans	*h* = −33→32
10830 measured reflections	*k* = −7→7
3026 independent reflections	*l* = −11→11

### Refinement

**Table:**

Refinement on *F*^2^	Secondary atom site location: difference Fourier map
Least-squares matrix: full	Hydrogen site location: inferred from neighbouring sites
*R*[*F*^2^ > 2σ(*F*^2^)] = 0.032	H atoms treated by a mixture of independent and constrained refinement
*wR*(*F*^2^) = 0.060	*w* = 1/[σ^2^(*F*_o_^2^) + (0.0215*P*)^2^] where *P* = (*F*_o_^2^ + 2*F*_c_^2^)/3
*S* = 0.89	(Δ/σ)_max_ < 0.001
3026 reflections	Δρ_max_ = 0.28 e Å^−^^3^
142 parameters	Δρ_min_ = −0.42 e Å^−^^3^
2 restraints	Absolute structure: Flack (1983), 1361 Friedel pairs
Primary atom site location: structure-invariant direct methods	Flack parameter: −0.015 (11)

### Special details

**Table:**

Geometry. All e.s.d.'s (except the e.s.d. in the dihedral angle between two l.s. planes) are estimated using the full covariance matrix. The cell e.s.d.'s are taken into account individually in the estimation of e.s.d.'s in distances, angles and torsion angles; correlations between e.s.d.'s in cell parameters are only used when they are defined by crystal symmetry. An approximate (isotropic) treatment of cell e.s.d.'s is used for estimating e.s.d.'s involving l.s. planes.
Refinement. Refinement of *F*^2^ against ALL reflections. The weighted *R*-factor *wR* and goodness of fit *S* are based on *F*^2^, conventional *R*-factors *R* are based on *F*, with *F* set to zero for negative *F*^2^. The threshold expression of *F*^2^ > σ(*F*^2^) is used only for calculating *R*-factors(gt) *etc*. and is not relevant to the choice of reflections for refinement. *R*-factors based on *F*^2^ are statistically about twice as large as those based on *F*, and *R*- factors based on ALL data will be even larger.

### Fractional atomic coordinates and isotropic or equivalent isotropic displacement parameters (Å^2^)

**Table:**

	*x*	*y*	*z*	*U*_iso_*/*U*_eq_	Occ. (<1)
Cu1	0	0.93278 (6)	0.79259 (5)	0.02848 (9)	
N1	−0.05624 (7)	1.1402 (3)	0.9220 (2)	0.0272 (4)	
O1	−0.05783 (6)	0.7137 (3)	0.69157 (18)	0.0362 (4)	
O2	−0.29533 (6)	0.7498 (3)	0.72580 (17)	0.0380 (4)	
O3	0	1.2442 (5)	0.5582 (4)	0.0721 (10)	
H1W	0.0259 (10)	1.360 (4)	0.562 (4)	0.086*	
C1	−0.32485 (8)	0.9589 (4)	0.8080 (5)	0.0432 (6)	
H1A	−0.3657	0.9321	0.7983	0.065*	
H1B	−0.3146	1.1216	0.754	0.065*	
H1C	−0.3142	0.9644	0.9274	0.065*	
C2	−0.23570 (8)	0.7593 (4)	0.7284 (2)	0.0300 (4)	
C3	−0.20813 (9)	0.5675 (4)	0.6291 (3)	0.0346 (5)	
H3A	−0.2302	0.4458	0.569	0.042*	
C4	−0.14955 (9)	0.5563 (4)	0.6192 (3)	0.0342 (5)	
H4A	−0.1325	0.4267	0.552	0.041*	
C5	−0.11349 (8)	0.7371 (4)	0.7087 (3)	0.0295 (4)	
C6	−0.14200 (7)	0.9290 (3)	0.8104 (4)	0.0279 (5)	
C7	−0.20319 (8)	0.9363 (4)	0.8174 (3)	0.0305 (5)	
H7A	−0.2214	1.0636	0.8837	0.037*	
C8	−0.11190 (8)	1.1207 (4)	0.9096 (2)	0.0298 (4)	
H8A	−0.134	1.2414	0.9705	0.036*	
C9	−0.03027 (8)	1.3274 (4)	1.0308 (3)	0.0274 (4)	
C10	−0.06016 (8)	1.4952 (4)	1.1327 (3)	0.0356 (4)	0.5
H10	−0.1003	1.4959	1.1326	0.043*	0.5
N2	−0.06016 (8)	1.4952 (4)	1.1327 (3)	0.0356 (4)	0.5
C11	−0.03003 (9)	1.6617 (4)	1.2347 (3)	0.0388 (5)	
H11	−0.0498	1.7778	1.3057	0.047*	

### Atomic displacement parameters (Å^2^)

**Table:**

	*U*^11^	*U*^22^	*U*^33^	*U*^12^	*U*^13^	*U*^23^
Cu1	0.02368 (14)	0.02800 (15)	0.03375 (16)	0	0	−0.0046 (2)
N1	0.0238 (8)	0.0287 (9)	0.0291 (9)	−0.0019 (6)	0.0017 (7)	−0.0028 (7)
O1	0.0257 (7)	0.0348 (8)	0.0480 (9)	0.0006 (6)	0.0017 (7)	−0.0130 (7)
O2	0.0247 (7)	0.0460 (8)	0.0433 (9)	−0.0026 (6)	−0.0047 (6)	−0.0076 (7)
O3	0.118 (3)	0.0387 (15)	0.0594 (18)	0	0	−0.0015 (15)
C1	0.0270 (9)	0.0522 (12)	0.0504 (16)	0.0024 (8)	−0.0035 (15)	−0.0011 (14)
C2	0.0248 (10)	0.0335 (10)	0.0316 (10)	−0.0030 (8)	−0.0023 (8)	0.0045 (8)
C3	0.0356 (12)	0.0313 (11)	0.0370 (11)	−0.0061 (9)	−0.0064 (9)	−0.0034 (11)
C4	0.0336 (11)	0.0279 (10)	0.0410 (12)	−0.0004 (9)	−0.0020 (9)	−0.0058 (10)
C5	0.0265 (10)	0.0284 (10)	0.0337 (10)	−0.0021 (8)	−0.0013 (9)	0.0023 (9)
C6	0.0263 (8)	0.0291 (8)	0.0283 (13)	−0.0029 (7)	−0.0004 (11)	−0.0010 (9)
C7	0.0292 (9)	0.0327 (9)	0.0296 (16)	0.0002 (8)	0.0014 (9)	−0.0012 (9)
C8	0.0280 (10)	0.0315 (11)	0.0299 (11)	−0.0002 (8)	0.0027 (8)	−0.0016 (8)
C9	0.0268 (10)	0.0287 (10)	0.0266 (10)	−0.0023 (9)	−0.0003 (8)	−0.0006 (9)
C10	0.0250 (9)	0.0442 (11)	0.0375 (11)	0.0022 (8)	0.0018 (8)	−0.0065 (9)
N2	0.0250 (9)	0.0442 (11)	0.0375 (11)	0.0022 (8)	0.0018 (8)	−0.0065 (9)
C11	0.0381 (11)	0.0387 (11)	0.0397 (13)	0.0083 (9)	0.0036 (8)	−0.0074 (10)

### Geometric parameters (Å, º)

**Table:**

Cu1—O1	1.9126 (14)	C3—C4	1.360 (3)
Cu1—O1^i^	1.9126 (14)	C3—H3A	0.93
Cu1—N1^i^	1.9561 (16)	C4—C5	1.425 (3)
Cu1—N1	1.9561 (16)	C4—H4A	0.93
Cu1—O3	2.416 (3)	C5—C6	1.421 (3)
N1—C8	1.297 (2)	C6—C7	1.419 (3)
N1—C9	1.410 (2)	C6—C8	1.427 (3)
O1—C5	1.302 (2)	C7—H7A	0.93
O2—C2	1.382 (2)	C8—H8A	0.93
O2—C1	1.419 (3)	C9—C10	1.356 (3)
O3—H1W	0.843 (16)	C9—C9^i^	1.402 (4)
C1—H1A	0.96	C10—C11	1.355 (3)
C1—H1B	0.96	C10—H10	0.93
C1—H1C	0.96	C11—C11^i^	1.391 (4)
C2—C7	1.364 (3)	C11—H11	0.93
C2—C3	1.400 (3)		
			
O1—Cu1—O1^i^	88.90 (8)	C4—C3—H3A	119.5
O1—Cu1—N1^i^	173.28 (7)	C2—C3—H3A	119.5
O1^i^—Cu1—N1^i^	93.47 (6)	C3—C4—C5	122.00 (19)
O1—Cu1—N1	93.47 (6)	C3—C4—H4A	119
O1^i^—Cu1—N1	173.28 (7)	C5—C4—H4A	119
N1^i^—Cu1—N1	83.50 (9)	O1—C5—C6	125.46 (18)
O1—Cu1—O3	94.28 (7)	O1—C5—C4	118.15 (18)
O1^i^—Cu1—O3	94.28 (7)	C6—C5—C4	116.40 (17)
N1^i^—Cu1—O3	91.82 (7)	C7—C6—C5	120.2 (2)
N1—Cu1—O3	91.82 (7)	C7—C6—C8	116.7 (2)
C8—N1—C9	121.37 (16)	C5—C6—C8	123.06 (16)
C8—N1—Cu1	125.63 (13)	C2—C7—C6	121.0 (2)
C9—N1—Cu1	112.97 (13)	C2—C7—H7A	119.5
C5—O1—Cu1	126.75 (12)	C6—C7—H7A	119.5
C2—O2—C1	116.66 (16)	N1—C8—C6	125.34 (18)
Cu1—O3—H1W	116 (2)	N1—C8—H8A	117.3
O2—C1—H1A	109.5	C6—C8—H8A	117.3
O2—C1—H1B	109.5	C10—C9—C9^i^	120.71 (11)
H1A—C1—H1B	109.5	C10—C9—N1	124.03 (17)
O2—C1—H1C	109.5	C9^i^—C9—N1	115.26 (10)
H1A—C1—H1C	109.5	C11—C10—C9	118.30 (18)
H1B—C1—H1C	109.5	C11—C10—H10	120.9
C7—C2—O2	125.62 (19)	C9—C10—H10	120.9
C7—C2—C3	119.34 (18)	C10—C11—C11^i^	120.99 (12)
O2—C2—C3	115.05 (17)	C10—C11—H11	119.5
C4—C3—C2	121.04 (18)	C11^i^—C11—H11	119.5
			
O1—Cu1—N1—C8	−6.06 (17)	C4—C5—C6—C7	−0.8 (3)
N1^i^—Cu1—N1—C8	179.97 (13)	O1—C5—C6—C8	−0.9 (4)
O3—Cu1—N1—C8	88.35 (16)	C4—C5—C6—C8	178.9 (2)
O1—Cu1—N1—C9	175.92 (12)	O2—C2—C7—C6	−179.7 (2)
N1^i^—Cu1—N1—C9	1.95 (15)	C3—C2—C7—C6	0.2 (3)
O3—Cu1—N1—C9	−89.67 (13)	C5—C6—C7—C2	0.5 (4)
O1^i^—Cu1—O1—C5	177.79 (13)	C8—C6—C7—C2	−179.27 (19)
N1—Cu1—O1—C5	4.09 (17)	C9—N1—C8—C6	−176.49 (19)
O3—Cu1—O1—C5	−88.00 (16)	Cu1—N1—C8—C6	5.6 (3)
C1—O2—C2—C7	7.1 (3)	C7—C6—C8—N1	178.4 (2)
C1—O2—C2—C3	−172.8 (2)	C5—C6—C8—N1	−1.4 (4)
C7—C2—C3—C4	−0.5 (3)	C8—N1—C9—C10	1.6 (3)
O2—C2—C3—C4	179.41 (18)	Cu1—N1—C9—C10	179.67 (16)
C2—C3—C4—C5	0.1 (3)	C8—N1—C9—C9^i^	−179.72 (13)
Cu1—O1—C5—C6	−1.6 (3)	Cu1—N1—C9—C9^i^	−1.61 (12)
Cu1—O1—C5—C4	178.60 (14)	C9^i^—C9—C10—C11	−0.2 (2)
C3—C4—C5—O1	−179.63 (19)	N1—C9—C10—C11	178.43 (17)
C3—C4—C5—C6	0.5 (3)	C9—C10—C11—C11^i^	0.2 (2)
O1—C5—C6—C7	179.4 (2)		

Symmetry code: (i) −*x*, *y*, *z*.

### Hydrogen-bond geometry (Å, º)

Please define Cg

**Table:**

*D*—H···*A*	*D*—H	H···*A*	*D*···*A*	*D*—H···*A*
O3—H1*W*···O1^ii^	0.84 (2)	2.19 (2)	2.933 (3)	146 (2)
C8—H8*A*···O2^iii^	0.93	2.57	3.330 (3)	139
C8—H8*A*···N2	0.93	2.49	2.844 (3)	103
C1—H1*B*···*Cg*^iv^	0.96	2.71	3.528 (4)	143

Symmetry codes: (ii) −*x*, *y*+1, *z*; (iii) −*x*−1/2, −*y*+2, *z*+1/2; (iv) −*x*+3/2, −*y*+2, *z*+1/2.
